# Synthesis of Jatropha-Oil-Based Polyester Polyol as Sustainable Biobased Material for Waterborne Polyurethane Dispersion

**DOI:** 10.3390/polym14183715

**Published:** 2022-09-06

**Authors:** Murni Sundang, Nur Sjanrah Nurdin, Sariah Saalah, Yamunah Jaibalah Singam, Syeed SaifulAzry Osman Al Edrus, Noor Maizura Ismail, Coswald Stephen Sipaut, Luqman Chuah Abdullah

**Affiliations:** 1 Chemical Engineering Programme, Faculty of Engineering, University Malaysia Sabah, Kota Kinabalu 88400, Sabah, Malaysia; 2 Higher Institution Centre of Excellence Wood and Tropical Fibre (HICoE), Institute of Tropical Forestry and Forest Products, Universiti Putra Malaysia, Serdang 43400, Selangor, Malaysia; 3 Department of Chemical and Environmental Engineering, Faculty of Engineering, Universiti Putra Malaysia, Serdang 43400, Selangor, Malaysia

**Keywords:** vegetable oil polyol, ring-opening, polyester polyol, waterborne polyurethane

## Abstract

The utilization of vegetable oil in the production of polymeric material has gained interest due to its proven ability to replace nonrenewable petroleum sources, as it is readily modified via chemical reaction to produce polyol and subsequently for polyurethane production. Jatropha oil (JO), a second-generation feedstock, is one of the suitable candidates for polyester polyol synthesis because it contains a high percentage of unsaturated fatty acids. In this study, jatropha-based polyester polyols (JOLs) with different hydroxyl values were successfully synthesized via a two-step method: epoxidation followed by oxirane ring-opening reaction. Ring-opening reagents; methanol, ethanol, and isopropanol were used to produce polyol with hydroxyl number of 166, 180, and 189 mg/KOH, respectively. All the synthesized JOLs exhibited a Newtonian to shear thinning behavior in the measured shear rate ranges from 10 to 1000 s^−1^ at 25 °C. The viscosity of a JOL ring-opened with methanol, isopropanol, and ethanol was 202, 213, and 666 mPa·s, respectively, at 20 °C and 100 s^−1^, which is within the range of commercially available polyols. Successively, the JOLs were reacted with isophorone diisocyanate (IPDI) to produce polyurethane prepolymer by utilizing 2,2-dimethylol propionic acid (DMPA) as an emulsifier. The prepolymer was then dispersed in water to produce a waterborne polyurethane dispersion. Colloidal stability of the jatropha-based polyurethane dispersions (JPUDs) were investigated by particle size analysis. A JPUD with a small particle size in the range of 6.39 to 43.83 nm was obtained, and the trend was associated with the soft segment of the polyol in the formulation. The zeta potentials of the JPUs ranged from −47.01 to −88.9 mV, indicating that all synthesized JPUs had high dispersity and stability. The efficient synthesis procedure, low cost, and excellent properties of the resulting product are thought to offer an opportunity to use jatropha oil as a sustainable resource for polyester polyol preparation.

## 1. Introduction

Fossil fuel depletion, environmental concerns, and petroleum price fluctuations are the main factors driving the increased worldwide interest in the production of biobased materials. Vegetable oil is one of the promising sources of biobased materials due to its abundancy and the fact that the production of vegetable oil has consistently increased worldwide for the past 30 years, reaching up to 240 percent. Furthermore, the interest in vegetable oil is also growing as a sustainability platform for the creation of environmentally benign functional polymers that are suitable for replacing existing petroleum-based polymers with comparable performance and cost [1].

Polyols can be categorized into polyester and polyether polyols, which are hydroxyl functional oligomers that are important precursors of PU. For the synthesis of biobased polyols, the most important vegetable oils are those that contain a high percentage of unsaturated fatty acid molecules in their triglyceride chain [2]. Jatropha oil (JO) was reported to contain about 78.9% unsaturated fatty acids and 21.1% saturated fatty acids [3,4]. Presently, polyester polyol derived from JO is used as a second-generation feedstock for polyurethane elastomers, alkyd resin, adhesives, and coatings [5,6,7,8,9]. JO is also suitable for biomaterial production because it addresses the main drawback of first-generation feedstock due to the presence of toxic ingredients called phorbol ester components that render it inedible [10]. As such, it is advantageous to use JO because it does not negatively impact the market for edible vegetable oil, which can also help reduce the use of other edible oils in materials production [3,4,11]. Hence, JO is a viable alternative to fossil fuel for the synthesis of biobased polyol and subsequently for polyurethane dispersion (PUD) production.

Traditional PU applied in the adhesives and coating industries is usually made up of 40–60 wt% volatile organic compounds (VOCs), which are harmful to the environment and cause damage to the human body [12]. This has led many researchers to explore more ecologically friendly polyurethane dispersion (PUD) by utilizing water as the primary solvent in environmentally benign coatings in the past year. As a result, PUD has become one of the most active and quickly emerging subfields of polyurethane research [13]. Furthermore, water is commonly considered a safe and cheap environmentally benign solvent due to its nontoxicity and abundant natural resources. Studies reported that PUD polymeric materials have good adhesion properties with most material due to their good dispersion in water and good film-formation ability [12]. Additionally, PUD possesses superior properties such as glossiness, clarity, a short drying rate, resistance to flexibility and temperature effects, wear resistance, nontoxicity, and nonflammability [14].

Researchers have successfully synthesized polyol from vegetable oil such as soybean oil, castor oil, rapeseed oil, palm oil, palmeri, vernonia, and carandol, and some of the products have been commercialized [15,16,17]. However, the synthesis of polyester polyol from jatropha oil is a new field and not widely explored. To date, limited studies have reported the effect of ring-opening reagents in synthesizing polyol from jatropha oil. Therefore, the objective of this research was to study the effect of ring-opening agents on the properties of jatropha-oil-based polyol.

**Scheme 1:**
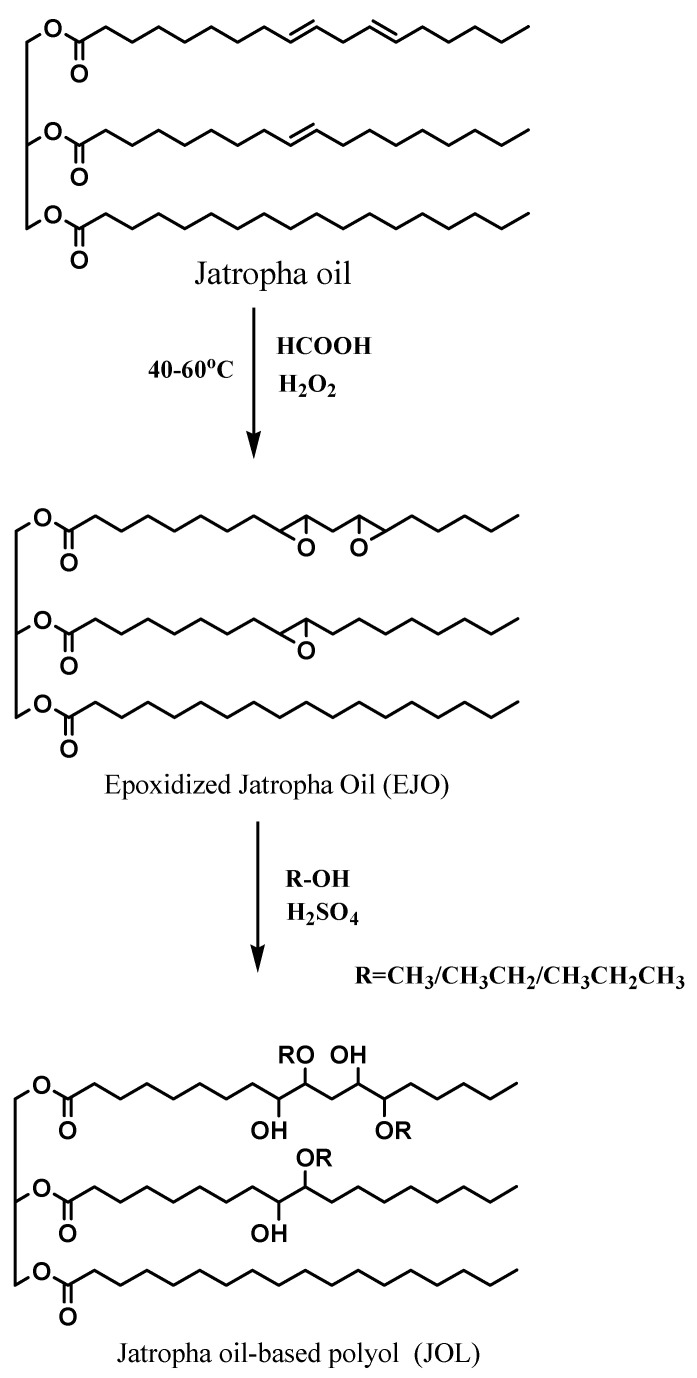
Synthesis of jatropha-oil-based polyester polyol (JOL).

In this research, we synthesized a series of polyols using a two-step method, namely epoxidation followed by an oxirane ring-opening reaction with different types of alcohol as the ring-opening reagent. Researchers have used many types of alcohol for the ring-opening reaction including methanol [6,18], ethanol [19], glycerol [20] and isobutanol [21]. Noor et al. (2022) also reported that an increase in the molecular weight of the ring-opening reagent for the ring-opening of epoxidized palm olein increased the viscosity of polyol [21]. Studies found the presence of hydroxyl groups in vegetable oils to enhance intermolecular interaction and further increase the viscosity of polyols [22]. In this study, we ring-opened the epoxidized oils with methanol, ethanol, and isopropanol to prepare the polyols, as illustrated in Scheme 1. These reagents differed in terms of molecular weight and the classification of the primary and secondary alcohol group. The jatropha-oil-based polyol was further used to synthesize polyurethane dispersion by reacting JOL with diisocyanate.

## 2. Materials and Methods

### 2.1. Materials

Crude jatropha oil with an acid value of 30.6 mg KOH/g was supplied by Biofuel Bionas Sdn Bhd, Kuala Lumpur, Malaysia. The main reagents for epoxidation, hydrogen peroxide 30% and formic acid, were supplied by Merck (Darmstadt, Germany) and Systerm, respectively. Sodium hydrogen carbonate (NaHCO_3_), sodium hydroxide (NaOH), ethyl methyl ketone (MEK), pyridine, and acetone were supplied by Merck, Germany. Alcohols used as the ring-opening agent were provided by Systerm. Magnesium sulfate (MgSO_4_) was procured from Sigma Aldrich. For PUD synthesis, isophorone diisocyanate (IPDI) with a NCO content of 38% was supplied by Aldrich. Dimethyl propionic acid (DMPA) acting as the ionic center and dibutyltin dilaurate (DBTDL) as the catalyst were supplied by Aldrich. All chemicals were reagent grade and used as received.

### 2.2. Synthesis of Jatropha-Oil-Based Polyester Polyol

#### 2.2.1. Epoxidation

The epoxidation of JO was carried out in a 1000 mL beaker equipped with a mechanical stirrer and thermometer. In order to keep the reaction temperature stable, the beaker was placed inside a water bath. Briefly, a mixture of jatropha oil and formic acid was placed in a 1000 L flask and heated to 40 °C under continuous stirring. Hydrogen peroxide (30%) was then added dropwise for 30 min before increasing the temperature to 60 °C. The reactant molar ratio of jatropha oil to formic acid to hydrogen peroxide used was fixed at 1:0.6:1.7 [4]. The mixture was allowed to react completely for 4 h, followed by cooling the mixture to room temperature. Then, the aqueous layer was discarded, and the remaining acid was eliminated by thoroughly washing the oil layer with an excessive amount of water. Magnesium sulfate was then added as a drying agent.

#### 2.2.2. Oxirane Ring-Opening

The reactions took place in a 1000 mL beaker equipped with a dropping funnel, magnetic stirrer, and thermometer. Methanol, ethanol, and isopropanol were used as the ring-opening reagent to ring-open the epoxidized jatropha oil (EJO). A calculated amount of ring-opening agent, water, and sulfuric acid catalyst was poured into the beaker and heated to the boiling points of various ring-opening agents under continuous stirring. EJO was then added to the mixture and the reactions proceeded for 30 min, followed by adding sodium bicarbonate to quench the reaction. After being cooled to room temperature, the deposit was discarded. The excess ring-opening agent and water were removed by vacuum distillation. Then, the three samples of jatropha-oil-based polyol (JOL) ring-opened using methanol (JOLM), ethanol (JOLE), and isopropanol (JOLI) were analyzed to determine their hydroxyl number according to ASTM D4274-99 (Test Method C-Reflux Phtalation).

### 2.3. Synthesis of Waterborne Jatropha-Oil-Based Polyurethane (JPU) Dispersions

**Scheme 2:**
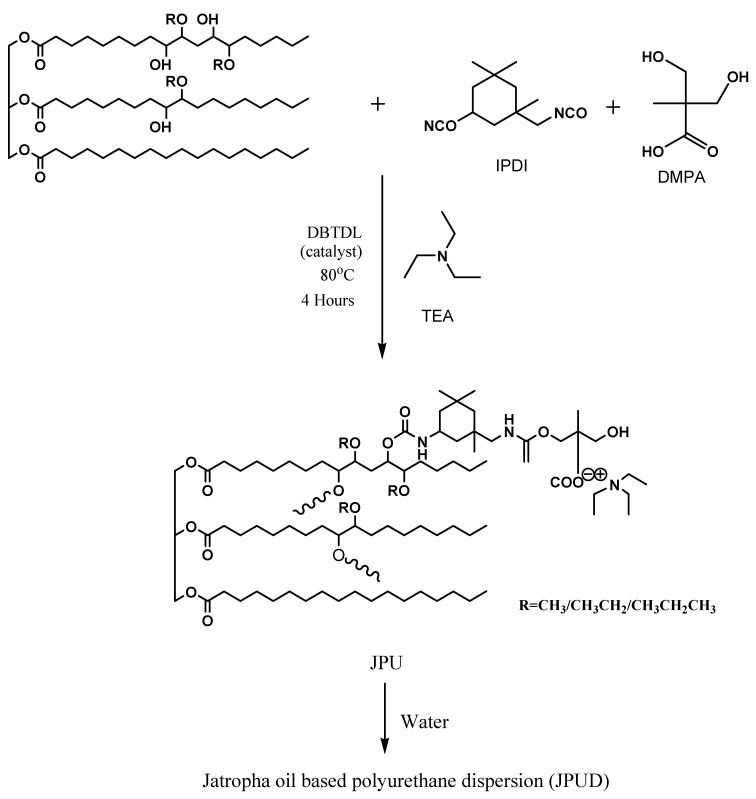
Synthesis of waterborne polyurethane dispersion.

JPU dispersions were synthesized according to a method reported by [23] with slight modification. Briefly, DMPA was dissolved in NMP and stirred at 50 °C. IPDI was then added dropwise followed by DBTDL as a catalyst. The reaction was performed at 80 °C, and MEK was used to reduce the viscosity of the prepolymer during the reaction. After three hours, the mixture was cooled to 50 °C and neutralized with TEA for 30 min. Finally, the PU prepolymer was dispersed in distilled water at a high stirring speed. Table 1 shows the formulation of the JPU dispersions. By fixing the DMPA content at 6.5 wt% and the molar ratio of NCO to OH at 1.0, JPUs with various hard segment content were obtained from three different types of polyols. The synthesis route of the polyurethane dispersion is shown in Scheme 2.

### 2.4. Characterizations

#### 2.4.1. Spectral Analysis

The functional groups of the JO, JOL, and JPU were verified using a Fourier transform infrared spectroscopy (FTIR) INVENIO^®^ Bruker model machine using the reflective attenuated total reflection (ATR) technique with OPUS 7.5 software (Bruker Optics, Ettlingen, Germany). Samples were directly placed on the diamond crystal, and the absorption spectra were recorded and analyzed within the range of 4000–450 cm^−1^. We recorded 32 scans with a nominal resolution of 2 cm^−1^.

#### 2.4.2. OH Number Determination

One of the most significant characteristics of polyols is their hydroxyl number, which indicates the number of hydroxy functional groups per gram of dry polyols. According to the ASTM D 4274-99 Test method C-phthalic anhydride reflux standard practice, the hydroxyl number for different jatropha-oil-based polyols was determined. This method uses a solution of phthalic anhydride in pyridine and is generally expected to quantitatively esterify all of the OH groups in a polyol. The hydroxyl number can also be used to calculate the polyol-equivalent weight using the following equation:

(1) $$\mathrm{Hydroxyl}\;\mathrm{equivalent}\;\mathrm{weight}=\frac{56.1\times 1000}{\mathrm{Hydroxyl}\;\mathrm{number}\;\left(\frac{\mathrm{mgKOH}}{g}\right)}$$

#### 2.4.3. Rheology

The rheological properties of the JOLs were analyzed using an Anton Paar Rotational Rheometer, model MCR 702e, equipped with double gap measuring geometries (DG26.7 SN85037) according to ISO/DIS3219-2. Rheology analysis was performed at steady-state flow modes to obtain the viscosity at 25 °C with the shear rate range set between 0 and 1000 s^−1^. The analysis of samples was also performed at various temperatures.

#### 2.4.4. Thermogravimetric Analysis (TGA)

The thermal stability of the jatropha oil and polyols was analyzed using a TA instrument, Q500 series (New Castle, DE, USA). The samples were heated from 25 to 600 °C, at a rate of 10 °C/min, under a nitrogen atmosphere.

#### 2.4.5. Differential Scanning Calorimetry

Differential scanning calorimetry (DSC) analysis for the jatropha oil and polyols was performed on a TA instrument, Q20 series (New Castle, DE, USA), according to ASTM D3418-03. The samples (5–10 mg) were heated from 25 to 100 °C at a rate of 10 °C/min to erase previous thermal history, cooled to −100 °C at a rate of 10 °C/min, and heated again to 150 °C at a rate of 10 °C/min under a nitrogen atmosphere.

#### 2.4.6. Particle Size and Zeta Potential

The particle size and zeta potential of the polyurethane dispersions were determined using a Zetasizer Nano-S (Malvern Panalytical Ltd., Malvern, UK). Prior to the analysis, 0.1 mL of the samples was diluted with 3 mL of distilled water. The particle size was measured at 173° using a backscattered configuration.

## 3. Results and Discussion

### 3.1. FTIR Analysis of JOL

**Figure 1:**
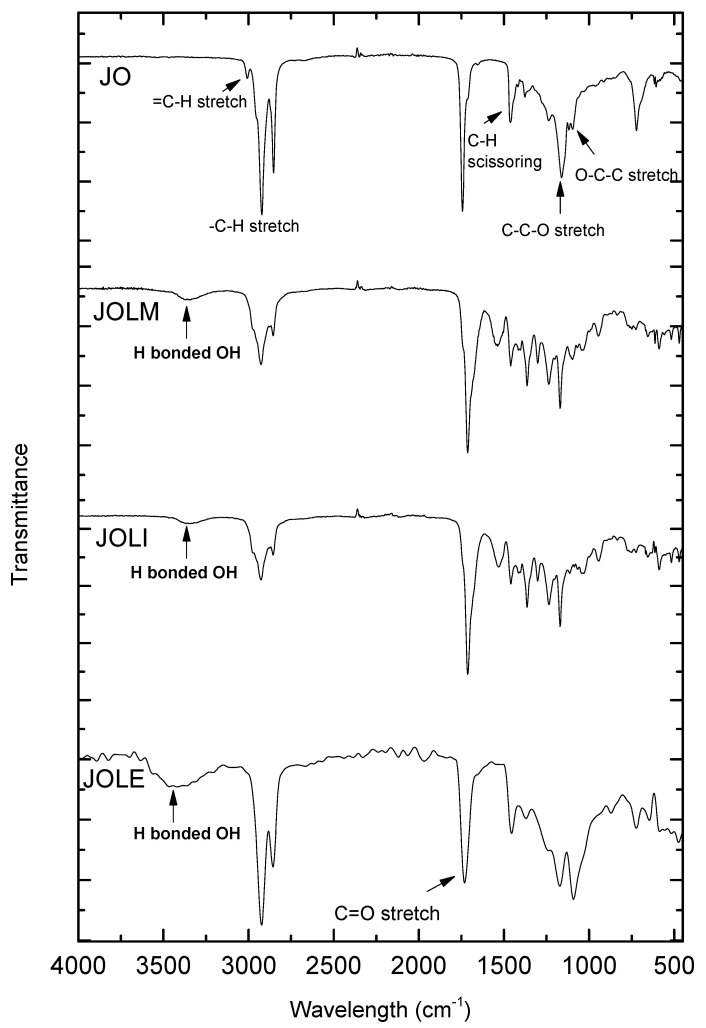
FTIR spectra of JO, JOLE, JOLM and JOLI.

Figure 1 shows the IR spectrum of JO, JOLE, JOLM and JOLI within the range of 4000–450 cm^−1^. The IR spectrum of JO shows that the absorption peak at 3010 cm^−1^ corresponds to bending vibration of the (C=)C–H double bonds [24], whereas the peaks at 2920 and 2850 cm^−1^ correspond to the antisymmetric and symmetric stretching vibrations of C–H in the CH_2_ and CH_3_ groups, respectively [25]. The strong peak at 1750 cm^−1^ was attributed to the ester C=O stretching vibration of the carbonyl groups present in the triglycerides in the oil [24]. Methylene C–H absorption is represented by the peak present at 1460 cm^−1^, while the peak at 1160 cm^−1^ represents ester C–O stretching. After undergoing the reactions of epoxidation and oxirane opening, the disappearance of the absorption peak at 3010 cm^−1^ in all JOL spectra indicated that the double bonds in the fatty acid of jatropha oil were fully reacted. The IR spectra of JOLE, JOLM, and JOLI showed broad peaks around 3600–3151 cm^−1^ indicating the existence of the OH group in all synthesized polyols.

### 3.2. Physicochemical and Rheological Properties of JOL

The OH number is an important characteristic of polyols, which determines the properties of the final PU product. Three samples of polyols were produced from different ring-opening reagents, namely methanol, ethanol, and isopropanol with OH numbers of 166, 180, and 189 mg KOH/g, respectively, as shown in Table 2. The value of the OH number is comparable to that of other vegetable-oil-based polyols synthesized using the same method as previously reported, such as soybean oil (135–200 mg KOH/g) [26], corn oil (143 mg KOH/g), linseed oil (147 mg KOH/g), and castor oil (163 mg KOH/g) [27].

**Figure 2:**
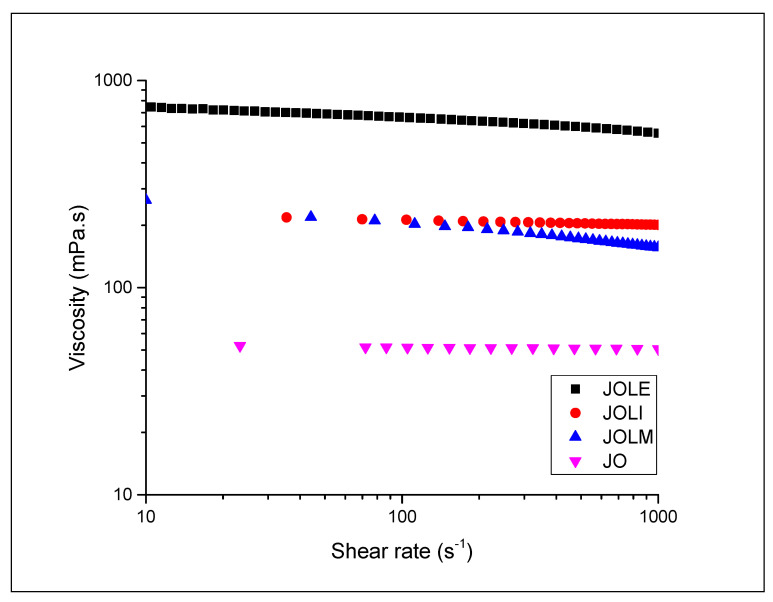
Viscosity of JO, JOLM, JOLI, and JOLE as a function of shear rate at 25 °C.

**Figure 3:**
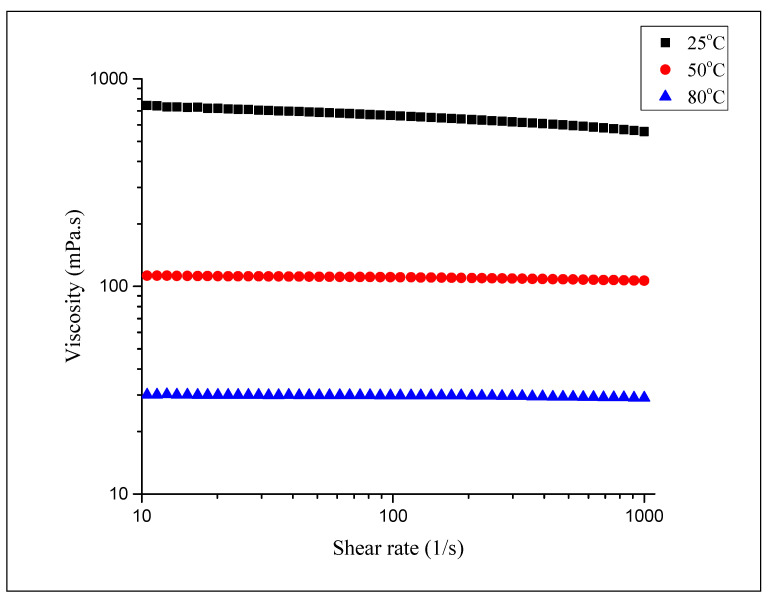
Viscosity of JOLE, as a function of shear rate at 25, 50, and 80 °C.

**Figure 4:**
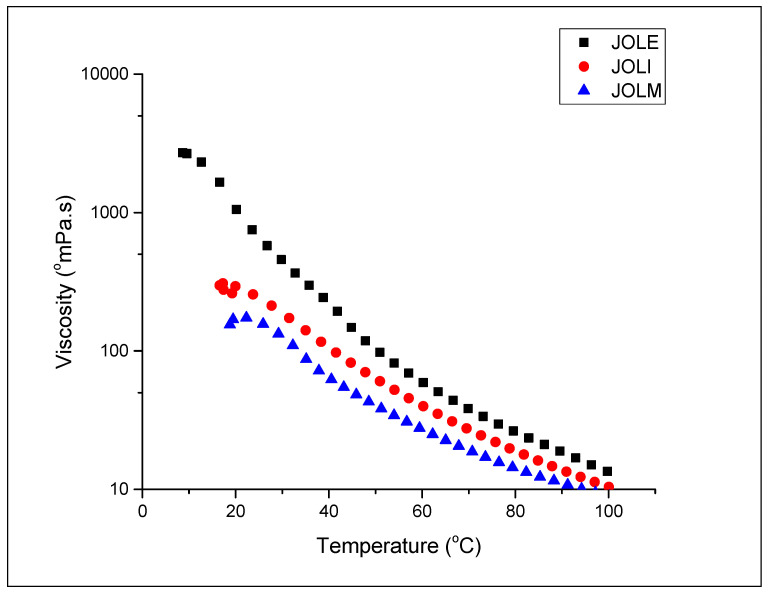
Viscosity of all JOLs at various temperatures.

We conducted a rheological analysis to determine the effect of the processing conditions, such as the shear rate and temperature influence, on fluid behavior. In the polyurethane industry, the viscosity of raw material determines the pumping process of polyol into the reaction unit to mix it with diisocyanate. Figure 2 illustrates the viscosity curves of JO, JOLI, JOLE, and JOLM against the shear rate. The observed constant viscosity of JO indicated that it exhibits Newtonian behavior in the measured shear rate ranges at a constant temperature of 25 °C. This finding that JO reflects Newtonian behavior is consistent with the findings of Mudri et al. [27]. Newtonian to shear thinning behavior can be observed in polyols at 25 °C as the viscosity decreases as the shear rate increases. This finding is consistent with the findings by Venkatesh and Jaisankar (2018), who produced polyols made of neem and jatropha oil that exhibited pseudoplastic non-Newtonian behavior [28]. Zhang et al. (2015) also reported that some of the samples of vegetable-oil-based polyol exhibited non-Newtonian behavior due to their greater molecular weight [29]. This behavior might be explained by the movement of molecules at the beginning of the flow field while rotating to change their direction. The viscosity remains constant when the molecules have aligned themselves to be parallel with the direction of the flow. At higher temperatures (50 and 80 °C), JOLE exhibited Newtonian behavior, where the viscosity remains constant as the shear rate changes, as shown in Figure 3. This might have been due to the lower degree of molecular orientation of the polyols at higher temperatures. Figure 4 shows that the increase in temperature reduced the viscosity of all the polyols. This is evidence that JOLs display Newtonian behavior during the manufacturing process because the typical industrial processing temperature for polyurethane dispersion is in the range of 60 to 90 °C [3,30]. Based on these data, we deduced that increasing the shear rate during the mixing process does not enhance the viscosity of the polyol when utilizing jatropha-oil-based polyol for polyurethane production. Thus, processing does not lead to the high viscosity of polyol, which should be avoided because high viscosity can cause problems [31].

The analysis on rheology properties indicated that the viscosities of polyol produced by different ring-opening agents (methanol, ethanol and isopropanol) increased in the following order: JOLM, JOLI, and JOLE, as shown in Table 2, at 25 °C and a shear rate of 100 s^−1^. The viscosity obtained for JOLM, JOLI, and JOLE was determined at 202, 213, and 666 mPa·s, respectively. This result correlated with that obtained by [32] using soybean oil at OH value ranges from 180 to 289 mg/KOH with viscosities from 567 to 1003 mPa at 45 °C. Furthermore, [22] also reported that the viscosity of modified vegetable oil increases with the presence of the hydroxyl group due to the intermolecular interactions. The viscosity of JOLs is comparable to that of the commercialized polyols, which range from 0.023 to 10 Pas [3,33].

### 3.3. Thermogravimetric Analysis

**Figure 5:**
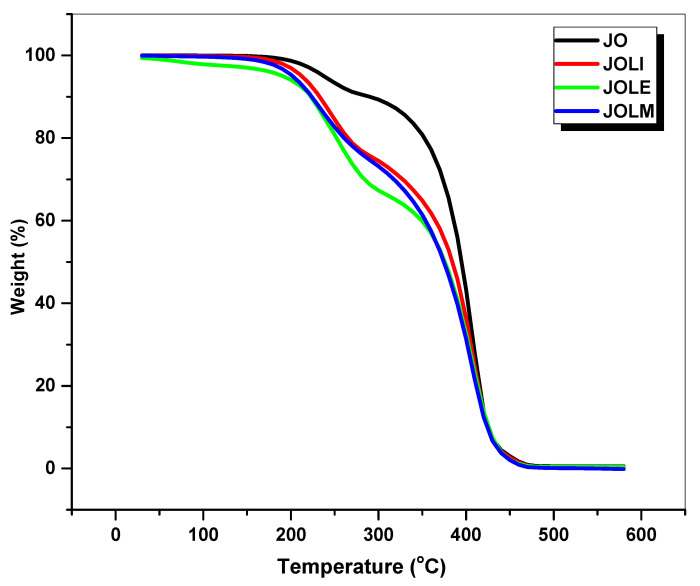
TGA thermograms of the JOL, JOLI, JOLE, and JOLM samples.

Thermogravimetric measurements were performed to obtain the thermal decomposition characteristics of JO and all of the prepared polyols. The resulting curves were plotted on the TG curve, as presented in Figure 5. Continuous loss in mass was examined as a function of time and temperature. We found that JO and the synthesized polyols evaporated or degraded among two ranges, the first from 170 to 280 °C and the second from 280 to 480 °C. JO shows higher oxidative stability compared with polyols produced due to the naturally occurring antioxidant component [34] such as tocopherols, complex phenols, and unsaturated fatty acids in the vegetable oil [35]. During the reactions of epoxidation and ring-opening, the antioxidant component of the oil was denatured and removed with water during washing; thus, the resulting polyols exhibited lower oxidative stability than the unprocessed JO. The weight loss curves of JO-derived polyols had nearly equal forms, and the differences in thermal stability seemed to be minimal as shown in Table 3.

### 3.4. Differential Scanning Calorimetry

**Figure 6:**
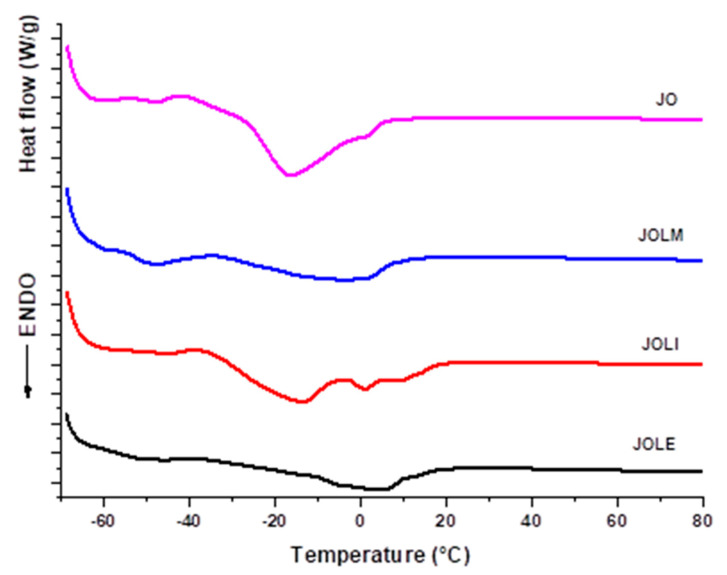
DSC plot for JO and polyols.

The DSC traces for the jatropha oil and polyols are displayed in Figure 6. A broad melting peak at −16.3 °C was observed in the jatropha oil. Multiple weak melting peaks between −13.1 and 5.8 °C were ascribed to different crystalline structures present in the polyols [36]. Because the highest melting peak located below room temperature as shown in Table 4, all polyols were still in liquid form at ambient conditions. Depending on the types of ring-opening agents, the liquid-to-greasy appearance of vegetable oil polyols has been reported in other studies [36,37].

### 3.5. FTIR Analysis of Jatropha-Oil-Based Polyurethane Prepolymer (JPU)

**Figure 7:**
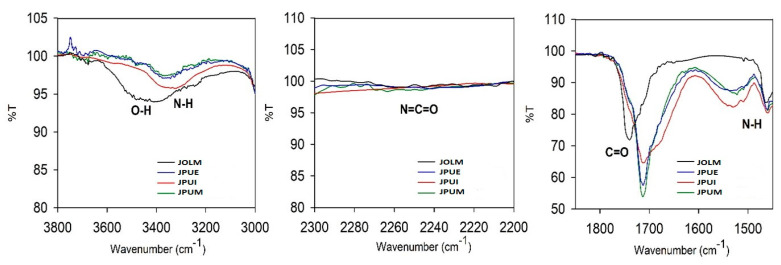
Essential functional groups in JPU samples.

The FTIR spectra for all JPUs in comparison with the polyol are presented in Figure 7. The FTIR spectra of JPUs confirmed the presence of functional groups characteristic of polyurethane. The wide absorbance of the –OH in JOLM, which was in the range of 3600–3300 cm^−1,^ disappeared in all JPUs [38] indicating that the OH group of polyol had fully reacted to form a hydrogen bond in the urethane group. The band around 3323 cm^−1^ appearing in all JPU samples corresponds to the free N–H stretching vibration of the urethane group. There were no significant peaks at the range between 2270 and 2300 cm^−1^, which was evidence of the absence of NCO groups in JPUs. This showed that the –NCO in IPDI completely reacted with the polyol. The absorption peaks at 1710 and 1540 cm^−1^ indicate C=O of urethane linkage and –NH_2_ deformations [19], respectively. The absorption bands at 1600 and 1510 cm^−1^ indicate the stretching and bending vibrations of C–N and N–H bonds, respectively, in urethane.

### 3.6. Colloidal Stability of JPU Dispersions

**Figure 8:**
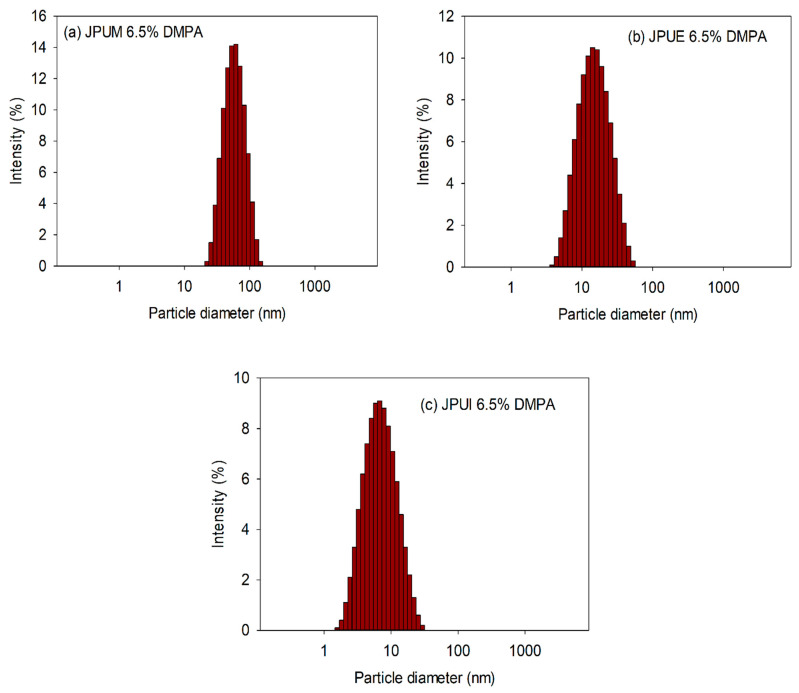
Particle size distribution and zeta potential of all JPU dispersions.

The particle size distribution of the JPU dispersions with different ring-opening agents is shown in Figure 8, while the particle size and zeta potential value are tabulated in Table 5. All the samples showed a unimodal distribution, which indicated homogeneity. Moving from JPUM, JPUE, to JPUI, the particle sizes tended to decrease from 43.83 to 6.39 nm. Having the same amount of DMPA as an emulsifier at 6.5 wt%, the decreasing trend could be explained by the decreasing amount of the polyols as the soft segment content, which is hydrophobic in nature.

A stable dispersion is commonly recognized by a lower particle size and higher zeta potential. Tielemans et al. (2006) proposed a stable colloid having a particle size of 100 nm or smaller [39]. In addition to stability, lower sizes of particles are suitable for spray applications, while larger sizes are usually associated with rapid drying [23,40]. The JPU dispersion showed zeta potential values ranging from −46.73 to −102.52 mV. The absolute value was higher than 30 mV, indicating that the particles tend to repel each other, and therefore are stable [41].

## 4. Conclusions

Jatropha-oil-based polyester polyol (JOL) was successfully synthesized by epoxidation and the oxirane ring-opening method, using ethanol, methanol, and isopropanol as the ring-opening agents. The JOLs were characterized by chemical and physical methods. The three JOLs were used to produce waterborne polyurethane dispersions, with 6.5 wt% DMPA content and a molar ratio of NCO/OH equal to one. The OH numbers of the JOLs were 166, 180, and 189 mgKOH/g for JOLM, JOLE, and JOLI, respectively. The polyols showed Newtonian to shear thinning behavior at 25 °C, and the viscosity was in the range of 156.2 to 556.6 mPa·s. Functionalization of the vegetable oil double bond to the hydroxyl group caused the viscosity to increase due to the hydrogen-bonded OH group in the JOLs. The viscosities of JOLE and JOLI were higher than that of JOLM, possibly due to the higher molecular weight of the alcohol used in the ring-opening steps. We also determined the thermal properties of the polyols. The resulting JPU dispersions exhibited the high absolute value of zeta potentials, ranging from −47.01 to −88.9 mV, and the particle sizes of 6.39 to 43.83 nm indicated that the dispersions were stable. Finally, jatropha oil’s cost effectiveness, sustainability, and capacity to produce greater yields hold particular promise for sustainable development.

## Figures and Tables

**Table 1:** The formulation of jatropha-oil-based polyurethane (JPU) dispersions.

Sample	Polyol	Molar Ratio			Percentage of DMPA (%)	^a^ Hard Segment (wt%)
		JOL	DMPA	IPDI		
JPUM	JOLM	1	0.53	1.53	6.5	44.14
JPUE	JOLE	1	0.48	1.48	6.5	45.64
JPUI	JPUI	1	0.45	1.45	6.5	47.27

^a^ Hard segment content, HS = Mass (IPDI + DMPA + TEA)/Mass (Polyol + DMPA + IPDI + TEA).

**Table 2:** Physicochemical properties of JO, JOLM, JOLE, and JOLI.

Sample	Ring-Opening Agent	OH No. (mg/KOH)	Viscosity at 25 °C (mPa·s) (At Shear Rate of 100 s^−1^)
JO	-	-	51
JOLM	Methanol	166 ± 11	202
JOLE	Ethanol	180 ± 5	666
JOLI	Isopropanol	189 ± 4	213

**Table 3:** The results of thermogravimetric analysis.

Sample	T_10%_, °C	T_max1_, °C	Mass Loss 1 (wt%)	T_max2_, °C	Mass Loss 2 (wt%)	Residue at 580 °C (wt%)
JO	289.65	242.36	9.75	409.20	89.73	0.50
JOLI	230.00	247.40	24.69	410.27	74.89	0.36
JOLE	220.00	254.27	33.35	409.18	65.65	0.38
JOLM	220.00	232.08	25.61	408.01	74.46	0.13

**Table 4:** DSC data for JO and polyols.

Sample	Peak 1 (°C)	Peak 2 (°C)
JO	−16.3	
JOLM	−3.5	
JOLI	−13.1	0.8
JOLE	5.8	

**Table 5:** The appearance, particle size, and zeta potential of JPUM, JPUE, and JPUI.

Dispersion	Soft Segment (wt%)	Color/ Appearance	Particle Size (nm)	Zeta Potential (mV)	PDI
JPUM	55.86	Milky white, opaque	43.83 ± 8.95	−64.67 ± 17.94	0.199
JPUE	54.36	Yellowish, transparent	12.90 ± 0.07	−47.01 ± 12.20	0.200
JPUI	52.73	Dark yellow, transparent	6.39 ± 0.04	−88.9 ± 13.62	0.292

## Data Availability

The data presented in this study are available on request from the corresponding author.
